# *Toxoplasma *seroprevalence in a rural population in France: detection of a household effect

**DOI:** 10.1186/1471-2334-9-76

**Published:** 2009-05-28

**Authors:** Emmanuelle Gilot Fromont, Benjamin Riche, Muriel Rabilloud

**Affiliations:** 1 Laboratoire de Biométrie et Biologie Evolutive, Equipe Ecologie Evolutive des Populations, UMR5558, CNRS, F-69622, Villeurbanne, France; 2 Laboratoire de Biométrie et Biologie Evolutive, Equipe Biostatistique Santé, UMR5558, CNRS, F-69310, Pierre-Bénite, France; 3 Université Lyon 1, F-69622, Villeurbanne, France; 4 Université de Lyon, F-69000, Lyon, France; 5 Service de Biostatistique, Hospices Civils de Lyon, F-69003, Lyon, France

## Abstract

**Background:**

*Toxoplasma gondii*, the agent of toxoplasmosis, has a complex life cycle. In humans, the parasite may be acquired either through ingestion of contaminated meat or through oocysts present in the environment. The importance of each source of contamination varies locally according to the environment characteristics and to differences concerning human eating habits and the presence of cats; thus, the risk factors may be determined through fine-scale studies. Here, we searched for factors associated with seropositivity in the population of two adjacent villages in Lorraine region, France.

**Methods:**

All voluntary inhabitants filled out a questionnaire and gave a blood sample. The seroprevalence was estimated globally and according to the inhabitants' ages using a cubic spline regression. A mixed logistic regression model was used to quantify the effect of individual and household factors on the probability of seropositivity.

**Results:**

Based on serological results from 273 persons, we estimated seroprevalence to be 47% (95% confidence interval: 41 to 53%). That seroprevalence increased with age: the slope was the steepest up to the age of 40 years (OR = 2.48 per 10-year increment, 95% credibility interval: [1.29 to 5.09]), but that increase was not significant afterwards. The probability of seropositivity tended to be higher in men than in women (OR = 2.01, 95% credibility interval: [0.92 to 4.72]) and in subjects eating raw vegetables at least once a week than in the others (OR = 8.4, 95% credibility interval: [0.93 to 72.1]). These effects were close to statistical significance. The multivariable analysis highlighted a significant seroprevalence heterogeneity among households. That seroprevalence varied between 6 and 91% (5^th ^and 95^th ^percentile of the household seropositivity distribution).

**Conclusion:**

The major finding is the household effect, with a strong heterogeneity of seroprevalence among households. This effect may be explained by common exposures of household members to local risk factors. Future work will quantify the link between the presence of oocysts in the soil and the seroprevalence of exposed households using a spatial analysis.

## Background

*Toxoplasma gondii *(*T. gondii*), the agent of toxoplasmosis, is one of the most widespread parasite species worldwide: in 2000, Tenter et al. estimated that one third of the human population might be parasitized [1]. In healthy humans,*T. gondii *infection is generally subclinical or mild. However, during pregnancy, the risk of materno-foetal transmission may have serious consequences for the foetus: abortion or congenital infection [2]. Most children with congenital toxoplasmosis have no symptoms at birth but are at risk of developing retinal diseases or neurological abnormalities later in life [3]. *T. gondii *may also cause severe encephalitis among immunosuppressed patients via acute infection or reactivation of a latent infection: encephalitis is the most frequent neurological infection among patients with acquired immunodeficiency syndrome [4].

Prevention of human toxoplasmosis relies on the knowledge of the disease epidemiology. However, the life cycle of *T. gondii *is complex: post-natal human infection may result from ingestion of tissue cysts contained in raw or undercooked meat from intermediate hosts or from ingestion or inhalation of oocysts shed by definitive hosts (felids) and disseminated in soil and water. Because virtually all mammals and birds may serve as intermediate hosts and because of the high resistance of *T. gondii *sporulated oocysts in the environment [5], possible sources of infection are multiple.

Given the multiplicity of those sources, the epidemiological situation of toxoplasmosis is spatially contrasted: incidence and prevalence values vary among countries and even among regions within countries [6,7]. The risk factors already identified differ also among studies. In a multicentre study in Europe, ingestion of tissue cysts has been estimated to represent 30 to 63% of the risk of infection, according to the locality, whereas contact with soil would represent 6 to 17%; finally, 14% to 49% of that risk remained unexplained [8]. Other studies found no relationship with ingestion of raw meat [9] or identified risk factors that were not clearly related to a known source of risk (e.g., the marital status) [7]. This variability reflects the fact that exposure to toxoplasmosis is probably variable at a local scale, depending on the climate, the agricultural methods, and on cultural and individual differences concerning eating habits or exposure to cats. Thus, searching for risk factors at a local spatial scale may be very informative.

The present survey on toxoplasmosis in the human population of two adjacent rural villages is a part of an analysis of toxoplasmosis dynamics. The aims were to provide a population-based estimate of *Toxoplasma *seroprevalence in a geographically-defined population, to describe the relationship between seroprevalence and factors like age and sex, and to search for other factors associated with prevalence at the scale of that population.

## Methods

### Study population

We studied the population of two villages, Barisey-la-Côte and Barisey-au-Plain, located in Lorraine region, Northeastern France. Since 1991, Barisey-la-Côte has been the site of a long-term survey on cat population biology and epidemiology [10,11], including a specific interest in toxoplasmosis. That rural site was considered appropriate for the study of the local variability of prevalence because rural area people often live in the same houses for long periods whereas, in urban areas, part of the inhabitants comes from other areas or countries and have specific risks [12]. A first information sheet was distributed via mailboxes. Then, from March to September 2004, two field investigators contacted directly each household. Consenting subjects aged at least 7 years old were asked to answer a questionnaire and give a blood sample. Each questionnaire was filled out by the investigator and given an anonymous number. Blood samples were collected by nurses after written consent was obtained from adult participants or minor's parents. The protocol has received the agreement of the Comité Consultatif de Protection des Personnes dans la Recherche Biomédicale (Centre Régional de Lutte Contre le Cancer Léon Bérard, Lyon (France) and the study was declared to the Commission Nationale de l'Informatique et des Libertés.

In the two villages, field investigators contacted directly 422 persons and failed to contact only 12 other identified individuals. Eighty-four percent of contacted persons accepted to fill out the questionnaire (353 answers) and 61% accepted to give a blood sample (256 samples). A previous positive serological status was more frequently a cause of refusal to provide a new blood sample than a negative status: 17/18 previously positive individuals versus 12/48 previously negative individuals. We included in the analysis the 17 previously positive subjects and thus analyzed the serological status of 273 subjects. The study involved 138 households. Twenty-five households (18%) had a single member, 75 (54.5%) two members, 21 (15%) three members, 11 (8%) four members, and 6 (4.5%) had 5 members.

### Questionnaire

The questionnaire sought information on: a) demographic characteristics: sex, age, localization of the accommodation; b) exposure to soil oocysts: number of years spent in the village, occupation related to agriculture, having a garden, time spent in the garden for gardening or other activities; c) exposure to contamination by food: use of bottled or tap water, frequency of meat consumption (beef, pork, poultry, lamb, mutton, goat, horse, rabbit, and game), frequency of consumption of undercooked beef, lamb, or pork, frequency of consumption of raw vegetables; d) contact with cats: presence of cats around the house, number of cats fed at home, cat litter in the house, total number of cats bred during life; e) history of toxoplasmosis: previous serological testing for toxoplasmosis and result, medical problem related to toxoplasmosis. The following classes were used to describe the frequency of meat or vegetable consumption: less than weekly, weekly, daily, more that once per day.

### Serological assay

Sera were kept frozen at -20°C and sent to the Department of Parasitology (Croix-Rousse hospital, Lyon, France) to be blindly tested for *Toxoplasma*-specific IgG and IgM using ELISA (Enzygnost Toxoplasmosis; Dade-Behring, Marburg, Germany) with a lysate of the parasite as antigen.

According to the results of the IgM serology, there were no recent infections in the study population. Among the 256 subjects who had an IgM serology, the titer was zero in 248 (97%) and between 3 and 5 (under the threshold of positivity) in the remaining eight subjects. Therefore, the seroprevalence was determined only on IgG serology results. Among the 256 subjects, 116 (45.3%) had a clearly negative result (titer under 10 IU), 29 (11.3%) an uncertain result (titer between 10 and 25 IU) and 111 (43.4%) a clearly positive result (titer over 25 IU, this being the threshold for positivity in the present study).

### Statistical analysis

The seroprevalence of *T. gondii *(with its 95% confidence interval) was estimated among the 273 subjects with known serological status. A cubic regression spline was used to represent the functional form of the link between seroprevalence and age.

A mixed logistic regression model with two levels (individual and household) was used to quantify the effects of individual and household factors on the probability of seropositivity. This model allowed us to take into account the fact that individuals from the same household would be exposed to a common environment and to quantify and explain the heterogeneity of seropositivity among households. The individual factors considered for this model were the demographic characteristics (location, age, and sex), the eating habits (consumption of raw vegetables, meat, or water from different origins), and the contact with cats and soil. To explain heterogeneity of seropositivity among households, these individual factors were aggregated to build household factors: the mean age of household members and various percentages of household members having the same eating habits or contact patterns with cats and soil. The parameters of the model were estimated with a Bayesian method. The analysis was carried out with WinBugs 1.4 (MRC Biostatistic Unit, Cambridge, UK) that uses Gibbs sampling.

## Results

Among the 273 subjects of the study, 61% (166/273) were living in Barisey-au-Plain, 56% were women (152/273), and 64% (175/273) were aged 20 to 60 years old. More than 90% of the inhabitants had gardens, consumed meat and raw vegetables at least once a week, and were in contact with cats around or in their houses (Table 1).

**Table 1:** Demographic characteristics of the population of Barisey-au-Plain and Barisey-la-Côte, and exposure to different sources of contamination.

Population characteristics	Number (%) N = 273
Barisey-au-Plain	166 (60.8%)
Barisey-la-Côte	107 (39.2%)
Age (years)	
< 20	30 (11.0%)
[20–40]	86 (31.5%)
[40–60]	89 (32.6%)
>= 60	68 (24.9%)
Women	152 (55.7%)
Men	121 (44.3%)
Occupation related to agriculture	30 (11.0%)
Having a garden	252 (92.3%)
Consumption of meat at least once a week	268 (98.2%)
Consumption of raw vegetables at least once a week	255/271 (94.1%)
Consumption of bottled water	69 (25.3%)
Consumption of tap water	121 (44.3%)
Presence of cats in or around the house	261 (95.6%)

**Figure 1:**
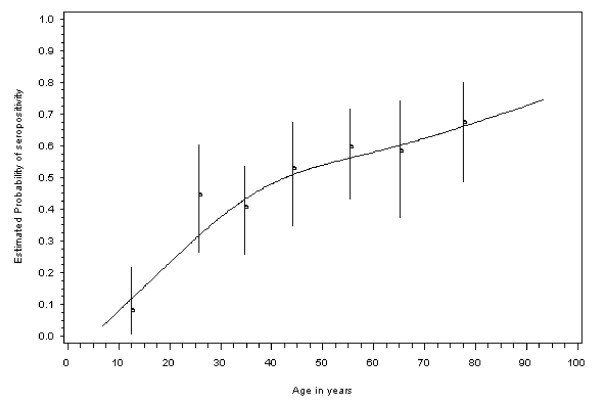
**Age-specific observed seroprevalence with 95% confidence intervals, in 7 age classes (<20, [20–30], [30–40], [40–50], [50–60], [60–70], and 70 years and over) and estimated probability of seropositivity depending on age (line)**.

The estimate of overall *Toxoplasma *seroprevalence was 47% (128/273, 95% CI: 41 – 53%). The functional form of the change of seroprevalence over age showed a strong increase between 7 and 40 years but a weaker increase after the age of 40 years (Figure 1). The estimated seroprevalence increased from about 2.5% at 7 years to about 50% at 40 years. At 95 years old, the estimated seroprevalence was about 75%.

After taking into account the effect of age, three factors tested in separate models were close to statistical significance: sex, consumption of raw vegetables at least once a week, and the number of cats bred during life (Table 2). These factors were further tested in a multivariable model (Table 3). *Toxoplasma *seroprevalence increased significantly with age up to 40 years old: the Odds of seropositivity were multiplied by 2.48 for each increase of 10 years in age. The progression of the probability of seropositivity was not statistically significant after 40 years old. Seroprevalence tended to be higher in men than in women with an Odds Ratio estimated at 2. This effect was close to statistical significance (Table 3). The Odds of seropositivity were multiplied by 8.4 in subjects eating raw vegetables at least once a week versus subjects who declared eating raw vegetables less often. Although only 16 subjects belonged to the latter category, the effect of consumption of raw vegetables was also close to statistical significance (Table 3). The number of cats bred during life tended to increase the seroprevalence, but this effect was weak, with an Odds Ratio very close to one that did not reach statistical significance (Table 3).

**Table 2:** Effect of demographic and exposure factors on seropositivity: results of the mixed logistic models adjusted on age.

Exposure factors	Odds Ratio adjusted on age – [95% CI*]
Barisey-la-Côte vs Barisey-au-Plain	0.85 [0.27; 2.76]
Men vs. women	1.83 [0.91; 4.22]
Occupation related to agriculture	1.89 [0.41; 9.98]
Having a garden	0.53 [0.09; 3.45]
Consumption of raw vegetables at least once a week	5.88 [0.86; 51.6]
Consumption of meat at least once a week	1.33 [0.03, 48.67]
Consumption of beef	1.08 [0.41; 2.70]
Consumption of lamb	2.13 [0.63; 7.29]
Consumption of mutton	0.95 [0.32; 2.95]
Consumption of pork	0.81 [0.25; 2.58]
Consumption of rabbit	3.42 [0.49; 26.9]
Consumption of game	0.79 [0.26; 2.64]
Consumption of bottled water	1.48 [0.41; 6.01]
Consumption of tap water	0.99 [0.34; 2.83]
Number of cats bred during life**	1.01 [0.95; 1.07]
Cat litter in the house	1.31 [0.32; 5.80]

* Credibility Interval

**Effect for one additional cat bred

After taking into account the mean age of household members in order to exclude a possible cohort effect (Table 3), the analysis revealed a statistically significant heterogeneity of the probability of seropositivity among households (variance estimated at 7 with a 95% credibility interval between 2.4 and 16.3). This strong heterogeneity among households corresponded to a seroprevalence varying between 6 and 91% (5^th ^and 95^th ^percentile of the household seropositivity distribution). None of the household factors we built to measure common eating habits or common exposures to cat or soil was able to explain that heterogeneity among households.

**Table 3:** Effect of demographic and exposure factors on seropositivity: result of the multivariable logistic mixed model

Exposure factors	Adjusted Odds Ratio [95% CI*]
Age until 40 years**	2.48 [1.29; 5.09]
Age after 40 years**	1.21 [0.76; 1.87]
Men vs. women	2.01 [0.92; 4.72]
Consumption of raw vegetables at least once a week	8.36 [0.93; 72.1]
Number of cats bred during life***	1.01 [0.95; 1.08]
Mean age of the family**	1.02 [0.98; 1.06]

* Credibility Interval

** Effect for a ten-year increase

*** Effect for one additional cat bred

## Discussion

In our specific rural setting, the overall seroprevalence and relationship with age were comparable to the values recently observed in neighboring geographical areas. In a recent population-based survey in the Netherlands, 40.5% of persons carried anti-*T. gondii *antibodies, the higher risks being those of people living in non-urbanized areas [7]. In France, the last perinatal survey indicated that 44% of women at the time of delivery were immune [13]. Although a standardization on age should be performed for direct comparison with other surveys, this seroprevalence estimate is higher than the values observed in other European countries: recent studies found a seroprevalence of 23.4% among blood donors living in rural areas in Slovakia [14] and of 23% among military personnel in the Czech Republic [15]. The relationship between seroprevalence and age is frequently observed and reflects the fact that the risk of toxoplasmosis infection remains present during whole life [14,15]. In the population-based survey in the Netherlands, the steepest rise in seroprevalence occurred in persons aged 25 to 44 years [7]. Here, we also show that the slope of the relationship between seroprevalence and age is steeper in young persons up to 40 years of age. This may reflect a higher risk in this age class, but this hypothesis cannot be disentangled from a birth-cohort effect.

There was a trend, although only close to statistical significance, towards a higher probability of seropositivity in men than in women. This result has not been found in other large population-based surveys [7,14,16] or, when found, the relationship seen in a univariate approach was no longer significant when other factors were considered [17]. However, a higher risk for men was also found in a previous study on a rural population in Illinois, USA [18] and in the middle and upper socio-economic classes in Brazil [19] suggesting that a difference exists but only in specific populations or settings. That higher risk for men was attributed either to contact with soil or to improper hygiene [17,18]. Thus, a difference between men and women might appear in populations with high exposure to soil. In the present study, men and women differed significantly as to the time spent gardening (4.5 h/week for men versus 2.8 for women); thus, as to exposure to soil.

*Toxoplasma *seroprevalence was not significantly associated with any of the factors related to meat consumption. In the univariate analysis, consumption of rabbit meat was the meat consumption factor with the highest estimated Odds Ratio, but this effect was far from statistical significance (Table 2), possibly because, in our population, few subjects never ate rabbit meat (only 27 subjects). This factor needs further investigation because it was found to be the major risk factor in a recent study carried out in the Czech Republic [15]. Besides the low sample size, the absence of any other factor concerning meat consumption may reflect the fact that this source of infection has become less important than others. Indeed, the latest evolutions of the agricultural methods have brought changes in the risk of infection of domesticated animals. For example, the seroprevalence in slaughter pigs is now under 8% in most countries; thus, the importance of pork meat as source of infection is probably declining [1]. The frequent consumption of frozen meat may also lower the risk of infection.

Consumption of raw vegetables was the only factor related to the eating habits that had a strong effect, close to statistical significance. This factor was also found associated with toxoplasmosis in Slovakia [14], in Norway (raw vegetables and fruits) [20], and in France (raw vegetables eaten outside home) [21]. Raw vegetables may be contaminated by soil oocysts or, in case of inappropriate handling, during meal preparation [21]. Checking and further investigating the link between contamination of raw vegetables and soil contamination will involve isolation of *T. gondii *oocysts from the soil and from locally available raw vegetables. A study conducted in an urban area with high densities of stray cat populations showed that high concentrations of oocysts can be detected in soil samples using real-time PCR [22].

Contrarily to the hypothesis that owning cats constitutes a significant risk factor, contact with cats, measured by the number of cats bred during life, was not found clearly associated with *Toxoplasma *seroprevalence. Actually, previous studies have found contradictory results about the link between contact with cats and the risk of toxoplasmosis. One study found that an increase in the number of seropositive cats trapped on the farm increased the risk of human infection [18]. Some have shown that owning a cat increased the risk of *T. gondii *seropositivity [15,21] while others found no association between daily contact with cats or the presence of cats in the environment and the risk of toxoplasmosis [8,20]. Kortbeek et al. [7] found only a weak association and concluded that owning a cat is not a risk factor whereas contamination of the environment with oocysts is. The present results suggest that the risk of contamination by oocysts may be more related to cats roaming outside shedding oocysts than to contact with household cats.

An important factor not reported before is the household effect: the probabilities of seropositivity for subjects belonging to the same household were closer than for subjects belonging to different households. This effect persisted after taking into account the mean age of household members; thus, it was not due to close ages within households. The heterogeneity, already found at country and region scales [6,7], was also found among households. An explanation of this similarity among members of the same household may be a common susceptibility due to genetic relatedness. However, that heterogeneity was also found in two-member households, most probably couples with no specific genetic relatedness (though this information was not checked). For example, among 57 two-member households in which the serological status of both members was known, 41 had concordant results and 16 discordant ones. The household effect resulted most probably from common risk factors within households. Common sources of exposure may be the household diet, the level of hygiene [21], or the contact with oocysts present in the soil around the house, which could be an important risk factor in this rural context [18]. In our study, none of the factors we aggregated at household level was able to explain heterogeneity among households. Because explaining the risk at the household level was not our primary aim, the household factors we built were probably not accurate measures of common exposures of households to the same risk factors. Except the mean age, those factors were built by aggregation of binary risk factors at household level; thus, the information they provided about the exposure to common risk factors was poor and their value could depend on the household size. We advocate that defining exposure at a household level should help highlighting relevant risk factors useful to predict individual risks and direct prevention.

## Conclusion

The major finding of our study was the household effect, with a strong heterogeneity of seroprevalence among households. This effect could be explained by common exposure of subjects sharing the same household to local risk factors. Our future work will be directed towards building quantitative factors measuring the presence of oocysts in the soil and towards quantifying the link between these factors and the seroprevalence of exposed households using a spatial analysis.

## Competing interests

The authors declare that they have no competing interests.

## Authors' contributions

EGF conceived of and coordinated the study. She participated in its design and drafted the introduction and the discussion of the manuscript. BR participated in the design of the study, performed the statistical analysis and helped to draft the manuscript. MR participated in the design of the study, supervised the statistical analysis and drafted the methods and the results of the manuscript. All authors read and approved the final manuscript.

## Pre-publication history

The pre-publication history for this paper can be accessed here:

http://www.biomedcentral.com/1471-2334/9/76/prepub
